# Percutaneous cryoablation in the management of spinal metastases: a comprehensive systematic review and meta-analysis

**DOI:** 10.1007/s11060-025-05064-3

**Published:** 2025-05-13

**Authors:** Mohammad Sadegh Fallahi, S. Farzad Maroufi, S. Parmis Maroufi, MirHojjat Khorasanizadeh, Leonardo José Monteiro de Macêdo Filho, Konstantinos Margetis, Daipayan Guha, Claudio E. Tatsui, Alireza Mansouri

**Affiliations:** 1 Neurosurgical Research Network (NRN), Universal Scientific Education and Research Network (USERN), Tehran, Iran; 2 Department of Neurosurgery, Tehran University of Medical Sciences, Tehran, Iran; 3 Department of Neurosurgery, Johns Hopkins University, Baltimore, MD USA; 4 Department of Neurosurgery, Medical College of Wisconson, Milwaukee, WI USA; 5 Department of Neurosurgery, Penn State Milton S. Hershey Medical Center, Hershey, PA USA; 6 Icahn School of Medicine at Mount Sinai, New York, NY USA; 7 Division of Neurosurgery, Hamilton General Hospital, McMaster University, Hamilton, ON Canada; 8 Department of Neurosurgery, The University of Texas MD Anderson Cancer Center, Houston, TX USA

**Keywords:** Percutaneous cryoablation, Spinal tumor, Spinal metastatic tumors

## Abstract

**Background:**

Minimally invasive techniques such as vertebroplasty, kyphoplasty, radiofrequency ablation, and stereotactic body radiotherapy have been widely used to manage spinal metastases. Among these, percutaneous cryoablation (PCA) has emerged as a promising option for local tumor control and pain management, offering targeted treatment with minimal damage to surrounding tissues. This systematic review and meta-analysis aimed to evaluate the efficacy and safety of PCA for spinal metastases.

**Methods:**

A systematic review was conducted using PubMed and Embase databases to identify studies that reported outcomes of PCA for spinal metastases. The reported radiologic, clinical, and complication outcomes were then combined and analyzed using meta-analytic methods including the calculation of pooled means and proportions, subgroup analysis, and meta-regression.

**Results:**

Eleven studies, including 229 patients, met inclusion criteria and were analyzed. Patients had a mean age of 61.8 years, with 60.6% being female. Breast (18.6%), lung (16.0%), and thyroid (8.0%) were the most common primary cancer sites. PCA was primarily conducted under general anesthesia (47.5%) and with CT/MRI guidance (93.9%). Local tumor control was achieved in 70.5% of cases over a mean follow-up of 12.6 months. Pain severity significantly decreased postoperatively, with a mean reduction of 4.5 points (*P* < 0.0001). Major and minor complication rates were 2.0% and 4.8%, respectively.

**Conclusions:**

PCA is an effective alternative treatment for spinal metastases, offering pain relief and local tumor control with low complication rates in appropriately selected patients. However, tumor location and patient age may influence treatment outcomes, underscoring the need for individualized treatment planning.

**Supplementary Information:**

The online version contains supplementary material available at 10.1007/s11060-025-05064-3.

## Introduction

Spinal metastases are common in cancer patients, causing severe pain and disability. Conventional external beam radiotherapy (cEBRT) is widely used but has limitations due to its toxicity to the spinal cord and, rarely, to surrounding organs such as the lungs and gastrointestinal tract. This necessitates dose fractionation, which can lead to radiation resistance in solid tumors [1]. Stereotactic body radiation therapy (SBRT) offers a more precise approach, reducing exposure to adjacent organs and allowing higher doses per fraction to overcome resistance. SBRT carries risks, such as vertebral compression fractures; however, this risk is potentially preventable with appropriate post-SBRT consolidation procedures [2]. Additionally, adequate tumor clearance from the spinal cord is essential for effectiveness [3]. Minimally invasive percutaneous ablation techniques, including alcohol, laser, microwave (MWA), and radiofrequency (RFA), have been explored for tumor destruction [4]. While beneficial for frail patients who cannot tolerate surgery, these methods pose risks such as vascular injury and neural damage [5, 6]. These challenges highlight the need for improved treatment strategies for spinal tumors.

Percutaneous cryoablation (PCA) is an emerging treatment used to destroy both superficial and deep-seated tumors by alternating cycles of freezing and thawing. PCA works by inducing direct and indirect cold-induced effects on the intra- and extra-cellular microenvironment [7]. These effects include cellular dehydration due to water movement [8], intracellular crystal formation leading to membrane and organelle damage, and apoptosis activation [9]. Additionally, PCA damages capillaries, causes thrombus formation, and increases inflammatory cytokines and edema through ice crystal formation [10]. A key advantage of PCA is real-time monitoring via computed tomography (CT) and magnetic resonance imaging (MRI), allowing for precise tumor targeting while preserving adjacent vital organs. It can be performed as an outpatient procedure without general anesthesia [11] and supports multi-probe capabilities similar to laser interstitial therapy (LITT) [12, 13]. Additionally, cryoablation excels in pain management by directly targeting nociceptive pathways through denervation of periosteal and tumor-associated nerves, leading to rapid and sustained reductions in pain scores and opioid dependence [14].

While PCA shows promise in treating spinal metastatic lesions, challenges remain regarding optimal patient selection and treatment protocols.

## Materials and methods

This systematic review followed the guidelines outlined in the 2020 update of PRISMA (Preferred Reporting Items for Systematic Reviews and Meta-Analyses) [15]. The study protocol was not registered in advance.

### Search strategy

A comprehensive search was conducted on PubMed and Embase from their inception to July 13, 2024. The search utilized the keywords “Cryoablation” AND “Spinal metastasis” along with relevant synonyms. The synonyms and complete search strategy is available in Supplementary Table 1. Additionally, the reference lists of the selected studies were reviewed to identify any relevant records that might have been overlooked.

### Study selection

Two independent reviewers (MSF and SFM) screened the identified articles by evaluating their titles and abstracts. The full texts of the selected records were retrieved and assessed for eligibility. Any disagreements were resolved through discussion, with the senior author (AM) making the final decision.

The inclusion criteria were as follows: (1) metastatic spinal tumors; (2) a minimum of three patients; (3) use of PCA as a primary, adjunctive, or salvage treatment; and (4) documentation of outcomes and complications related to PCA for spinal metastases. The exclusion criteria were: (1) studies involving primary spinal lesions, (2) use of other cryosurgery techniques, (3) lack of reported outcomes or complications, (4) studies not published in English, (5) non-original research and case reports, and (6) studies with fewer than 3 cases.

### Data extraction

Two independent reviewers (MSF and SFM) conducted data extraction from the selected studies, employing a standardized Microsoft Excel datasheet. The data collected included study characteristics (author, publication year, study design), patient demographics, tumor features, treatment specifics, local tumor control, pain severity scale and complications. Any disagreements were resolved through collaborative discussion.

### Risk of bias assessment

The Joanna Briggs Institute’s Case Series Checklist [16] was used to evaluate the risk of bias in the included studies, as it is specifically designed for non-randomized, observational studies, that often lack a control group. Two independent reviewers (MSF and SPM) assessed each article and disagreements were addressed through discussion with input from the senior author (AM) when necessary.

### Statistical analysis

A meta-analysis was conducted using R Studio (version 4.1.2) and the “meta” package. Means and standard deviations were calculated using the method proposed by Hozo et al. [17] Proportions and means were combined using the inverse variance and DerSimonian-Laird methods. Given the heterogeneity of PCA procedures across studies, a random-effects model was employed to report effects. Meta-regression analysis was performed to identify influential factors, while funnel plots were visually inspected for publication bias. A p-value less than 0.05 was considered statistically significant.

## Results

**Fig. 1:**
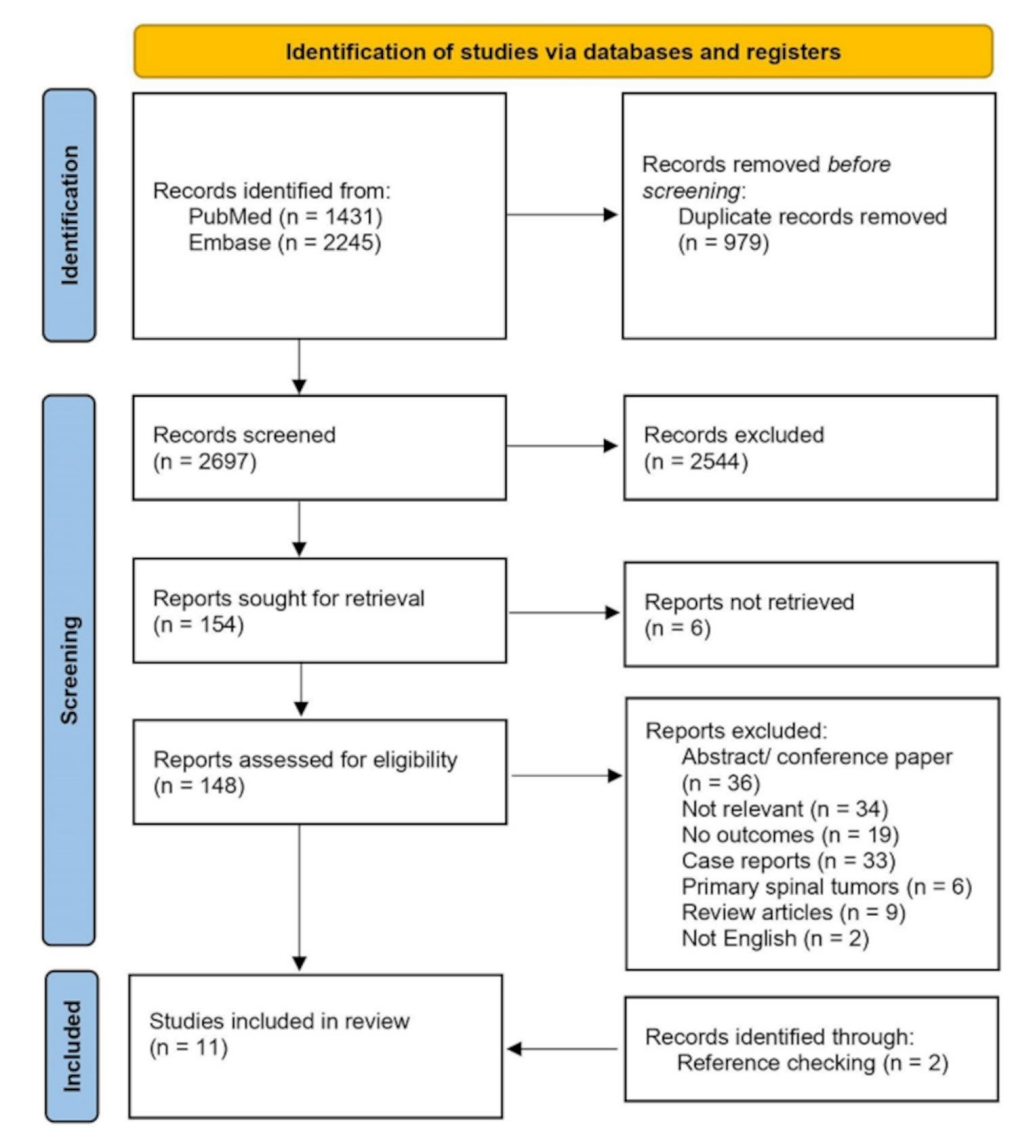
Flow diagram of screening process

The search strategy resulted in 3,676 records, out of which 979 were duplicates. After screening the titles and abstracts, 140 articles were selected for a full-text review. Nine articles met the eligibility criteria and were included in the quantitative analysis (Fig. 1).

Additionally, two more records were found through reference checking [13, 18–27].

Out of the 11 studies analyzed, 10 were retrospective, and one was a prospective trial. Four studies were conducted in the United States, four in France, two in Italy, and one in Switzerland. In total, these studies involved 229 patients diagnosed with spinal metastases (Table 1). Additional details—such as prior treatments, anesthesia methods, and adjuvant therapies—are provided in Supplementary Table 3.

**Table 1:** Characteristics of included studies

Author, year	Study design	Country	*N* of patients	Mean age	Metastasis origin	Tumor location in spine	PCA device	PCA Probe	Pain Scale	Last follow-up (months)
Cazzato et al., 2022	Retrospective	France	74	61	Breast (18), Lung (17), Kidney (7), Thyroid (5), Colorectal (5), Other (13)	Cervico-thoracic (58), Lumbar (35), Sacrum-Coccygeal (12)	SeedNet	IceSphere, IceRod	NRS	17.2 ± 20.2
Gravel et al., 2022	Retrospective	France	3	NR	NR	Thoracic (2), Lumbar (1)	VisualICE	IceSphere	NR	NR
Autrusseau et al., 2021	Retrospective	France	41	59.7	Breast (16), Lung (9), Kidney (5)	Thoracic (33), Lumbar (13)	NR	IceSeed, IceSphere, IceRod	NRS	Median 16.5 ± 23.2
Moses et al., 2020	Prospective	United States	14	54.5	Lung (4), Breast (3), Kidney (3), Tonsil (1), Thyroid (1), Rectum (1), Melanoma (1)	Thoracic (10), Sacral (2), Lumbar (1), Cervical (1)	SeedNet	IceSeed	VAS	Median 7.03 (range 0.82–25.38)
Gravel et al., 2019	Retrospective	France	39	NR	Thyroid (16), Breast (7), Lung (5), Paragangelioma/ Pheochromocytoma (3), Other (8)	NR	VisualICE	IceSeed, IceSphere, and IceRod	NR	12
Gallusser et al., 2019	Retrospective	Switzerland	4	68.5	Breast (1), Lung (1), Tonsil (1)	Thoracic (2), Lumbar (1), Sacrum (1)	NR	NR	NRS	14.75 ± 16.98
Motta et al., 2017	Retrospective	Italy	11	68.8	Breast (4), HCC (2), Renal (1), prostate (1), Bladder (1), Ovarian (2)	Lumbar (3), Sacrum (6), Thoracic (2)	NR	IceRod, IceSeed	VAS	Median 7.4 (range 3–13)
McArthur et al., 2017	Retrospective	United States	3	60.3	Esophageal (2), HCC (1)	Thoracic (1), Lumbar (1), Sacrum (1)	NR	PERC-15, PERC-24	NR	22.99 ± 30.48
Tomasian et al., 2016	Retrospective	United States	14	49.7	Lung (4), CRC (3), Follicular thyroid carcinoma (2), head and neck squamous cell carcinoma (1), pancreatic adenocarcinoma (1), epithelioid hemangioendothelioma (1)	Lumbar (14), Thoracic (8), Sacrum (6), Coccyx (2), Cervical (1)	NR	Endocare Perc-15 and Perc-17, Galil cryoprobes	NRS	Median 10 (range 1–24)
Guenette et al., 2016	Retrospective	United States	3	55.7	Lung (1), Leiomyosarcoma (1), Adenoid cystic (1)	Thoracic (2), Cervical (1)	SeedNet	IceSphere, IceSeed, or IceRod	NR	Range 1–22
Masala et al., 2013	Retrospective	Italy	23	73.3	NR	Thoracic (17), Lumbar (6)	NR	IceRod, IceSeed	VAS	6

Abbreviations: NR: Not reported; NRS: Numeric Rating Scale; VAS: Visual Analogue Scale

### Baseline characteristics

The mean age of the patients was 61.8 years, with 60.6% being female. The origins of the metastases included breast (18.6%), lung (16.0%), and thyroid (8.0%), with other primary sites accounting for 45.0% of the tumors, including kidneys, ovaries, and colon. The most common tumor locations were cervicothoracic (54.8%) and within the vertebral body (47.3%). Additionally, 36.6% of the tumors had epidural involvement, and 60.6% of the lesions had previously received radiation therapy (RT). The average pre-operative pain severity score reported by patients was 7.0 (Table 2).

**Table 2:** Meta-analytic details of baseline characteristics, PCA details, outcomes, and complications

	*N* studies	*N* patients/ lesions	Value (95% CI)	I^2^	Tau	H
**Baseline characteristics**						
Age, Years	9	187	61.82 (56.56–67.08)	96.0%	7.16	5.01
Females, %	10	225	60.58 (49.70-71.47)	58.3%	0.12	1.55
*Metastasis origin*						
Breast, %	9	203	18.64 (7.71–29.57)	71.6%	0.13	1.88
Lung, %	9	203	15.99 (10.96–21.03)	45.2%	0.07	1.35
Thyroid, %	9	203	8.03 (0.48–15.57)	73.7%	0.08	1.95
Other, %	9	203	44.98 (32.85–57.11)	62.4%	0.13	1.63
Tumor size, cm	6	84	2.48 (1.85–3.11)	86.0%	0.65	2.68
*Tumor localization*						
Cervico-thoracic, %	10	243	54.77 (40.77–68.76)	75.2%	0.17	2.01
Lumbar, %	10	243	26.21 (17.37–35.06)	47.6%	0.09	1.38
Sacro-coccygeal, %	10	243	10.72 (3.08–18.37)	75.3%	0.08	2.01
*Tumor location in vertebra*						
Body, %	5	208	47.33 (13.66–81.01)	97.5%	0.37	6.35
Arch, %	5	208	36.48 (13.12–59.85)	95.1%	0.25	4.50
Both, %	5	208	8.79 (0.00- 18.54)	86.3%	0.09	2.71
Epidural involvement, %	4	160	36.59 (6.52–66.65)	96.2%	0.30	5.15
Previous radiation therapy, %	6	122	60.60 (11.98- 1.00)	99.3%	0.60	11.64
Pain severity score	8	174	6.98 (5.75–8.21)	97.1%	1.73	5.86
**Procedural details**						
General anesthesia, %	10	239	47.59 (18.35–76.83)	98.4%	0.46	7.82
CT/MRI guidance, %	9	200	93.93 (84.88–100.00)	88.0%	0.11	2.89
*Protective measures*						
Hydrodissection, %	5	216	24.74 (0.00–52.00)	97.8%	0.30	6.78
Carbodissection, %	5	216	42.58 (16.42–68.73)	95.5%	0.29	4.70
Thermal monitoring, %	5	231	42.87 (0.00-88.94)	99.3%	0.52	11.90
Neural monitoring, %	5	231	11.88 (2.02–21.73)	93.4%	0.10	3.89
Additional vertebroplasty/ cementoplasty, %	7	275	55.38 (23.24–87.53)	98.3%	0.42	7.72
**Outcomes**						
Follow up, months	7	161	12.55 (9.06–16.04)	72.4%	3.44	1.90
Local tumor control, %	8	136	70.51 (52.64–88.38)	83.9%	0.21	2.49
*Post-operative pain severity score*						
1-day	3	116	3.70 (3.37–4.02)	23.3%	0.15	1.14
1-month	4	139	2.40 (2.13–2.66)	47.5%	0.27	1.38
Last follow-up	8	174	2.62 (2.20–3.04)	68.1%	0.48	1.77
*Complications*						
Major, %	10	225	2.03 (0.00- 4.24)	0.0%	0.00	1.00
Minor, %	10	225	4.81 (1.78–7.84)	6.9%	0.01	1.04

### PCA details

Patient selection criteria for PCA varied across studies. Most commonly, patients included had oligometastatic disease resistant or recurrent after conventional treatments such as first line chemotherapy and RT. Additionally, patients who declined or were not suitable for open surgery were often considered for PCA (Supplementary Table 2). Overall, 47.5% of procedures were performed under general anesthesia, and 93.9% were conducted with CT or MRI guidance. The most common protective measure used were thermal monitoring (42.9%) and carbodissection (42.6%), followed by hydrodissection (24.7%) and neural monitoring (11.9%). Cement consolidation of the vertebral body was performed in 55.4% of patients (Table 2).

### Outcomes

The mean follow-up duration for patients was 12.6 months. During this period, local tumor control (defined as the absence of tumor growth compared to baseline CT or MRI imaging, or absence of radiotracer uptake within or around the ablation zone on PET-CT at the last follow-up.) was achieved in 70.5% of cases (Table 2). Meta-regression analysis revealed a significant negative correlation between the presence of tumors in the vertebral body and local tumor control rates. Additionally, age demonstrated a trend toward a negative association with tumor control rates (*P* = 0.055) (Table 3).

**Table 3:** Meta-regression details for different factors affecting local tumor control and pain reduction

	No of studies	Estimate	SE	*P* value	R2
**Local tumor control**					
Year of publication	8	0.0170	0.0431	0.6937	0.00%
Age	6	-0.0291	0.0151	0.0547	31.07%
Female sex	7	0.2809	0.9969	0.7781	0.00%
Size	4	-0.1961	0.2555	0.4428	0.00%
Cervico-thoracic location	7	-0.5106	0.5815	0.3799	0.00%
Body of vertebra	5	-0.5062	0.2160	0.0191	75.78%
Previous RT	3	-0.5870	0.7994	0.4627	0.00%
Breast origin	7	-0.5332	0.6663	0.4236	20.50%
Lung origin	7	0.3362	1.0303	0.7441	31.49%
General anesthesia	7	-0.3728	0.2312	0.1069	16.01%
Vertebral augmentation	6	0.2139	0.3697	0.5628	0.00%
**Pain reduction**					
Year of publication	8	-0.3014	0.1366	0.0273	48.23%
Age	8	0.0395	0.0755	0.6009	0.00%
Female sex	7	2.9379	2.3220	0.2058	26.66%
Size	4	-0.9900	0.6089	0.1040	41.74%
Cervico-thoracic location	8	-1.3077	2.6883	0.6267	0.00%
Body of vertebra	4	3.2299	0.9416	0.0006	88.61%
Previous RT	5	1.0781	3.9902	0.7870	0.00%
Breast origin	7	-2.6532	3.4090	0.4364	0.00%
Lung origin	7	-5.7048	3.8183	0.1352	9.54%
General anesthesia	7	-1.3460	0.8715	0.1225	22.80%
Vertebral augmentation	5	2.4487	2.0714	0.2372	6.26%

The mean pain scores were 3.7 at 1 day, 2.4 at 1 month, and 2.6 at the last follow-up (Table 2). Pain significantly decreased from the pre-operative state to 1-day post-procedure (mean difference of -3.2 *p* < 0.0001) (Supplementary Fig. 1A) and further decrease by 1.3 points between the 1-day and 1-month follow-ups (*P* < 0.0001) (Supplementary Fig. 1B). Scores remained stable from the 1-month to the last follow-up (*P* = 0.29) (Supplementary Fig. 1C). Overall, there was a significant reduction in pain scores of 4.5 points from pre-operative to last follow-up (*P* < 0.0001) (Supplementary Fig. 2). Meta-regression analysis showed that higher rates of tumors located in the vertebral body were positively associated with pain reduction (*P* < 0.001), while the year of publication showed a negative correlation (*P* = 0.027) (Table 3).

### Complications

Major complications were defined as those classified as CTCAE grade ≥ 3 or as major according to the Society of Interventional Radiology (SIR) adverse event (AE) classification system. Minor complications were defined as CTCAE grades 1–2 or as minor according to the SIR AE classification. The pooled rates of complications following PCA were 2.0% for major and 4.8% for minor. Major complications included a third-degree atrioventricular block [18], left lower extremity weakness [18], persistent paraparesis [22], Tako-tsubo cardiomyopathy [22], and intraoperative cardiac arrhythmia [20]. Minor adverse events totaled 17 including nine transient nerve root radiculopathies [13, 18, 22, 24], two brachial plexus injuries [18, 20], two post-ablation pain requiring analgesics [18, 20], one short-term neuropraxia [25], one left quadriceps weakness [18], a broken biopsy needle in the T1 vertebral body [18], and one distended bladder requiring catheterization [20].

### Risk of bias

Most of the studies analyzed had a low risk of bias (Supplementary Table 4). Only two studies were found to have a high risk of bias, primarily due to insufficient reporting of outcomes.

## Discussion

Spinal metastases are treated with systemic therapies, surgery, and radiation. While surgery provides decompression and stabilization, it is invasive and unsuitable for frail patients with advanced disease. In such cases, PCA offers a minimally invasive alternative for targeted tumor destruction, with real‑time monitoring of the iceball to control its margins and limit injury to surrounding critical structures. PCA provides rapid pain relief, shorter hospital stays, and quicker return to oncological treatment [28]. Our analysis shows that PCA achieves adequate tumor control and effective pain reduction with low complication rates, primarily minor and reversible. These findings suggest PCA is a promising option for managing spinal metastases in carefully selected patients.

### PCA procedure, devices, and protective measures

PCA procedure can be performed under general anesthesia, conscious sedation, or local anesthesia [13]. Various imaging modalities such as CT scan and MRI can be used to monitor the formation of the frozen area at the tip of the cryoprobe confirming destruction of the tumor and preservation of surrounding tissue. Protective measures such as hydro-dissection, carbo-dissection, thermal and neuro monitoring are described as adjuncts to reduce the incidence of complications during the procedure. Hydro-dissection involves injecting warmed 5% dextrose to create a barrier between the tumor and adjacent structures, shielding them from extreme cold [13]. Carbo-dissection uses injection of carbon dioxide (CO2) in the epidural space to act as a cold barrier to protect the spinal cord [19]. Neuro-monitoring using somatosensory evoked potentials (SSEP) and motor evoked potentials (MEPs) have been used to minimize the risk of neural injury during PCA performed under general anesthesia [29–31].

Carbo-dissection poses challenges due to gas redistributing within the epidural space instead of forming a layer between the tumor and surrounding structures, especially in epidural tumors compressing the dura where space is limited [32]. Hydrodissection also has drawbacks, including the need for precise needle placement, which can be difficult in osteolytic lesions with damaged bone structure. Additionally, it may be ineffective in patients with postoperative adhesions or those who have undergone radiotherapy. Neuro-monitoring techniques, while valuable for detecting impending injury, offer reactive rather than proactive feedback. This may delay intervention until after irreversible damage has occurred, highlighting their role as a supplemental rather than primary protective measure. Performing PCA under imaging guidance allows for control of iceball margins; however, the resulting temperature drop can extend to the cortical surface, potentially injuring the spinal cord or nerve roots [33]. Further comparative research on these techniques in spinal tumor cases is needed to determine the most effective and safest approach.

### Outcomes

**Fig. 2:**
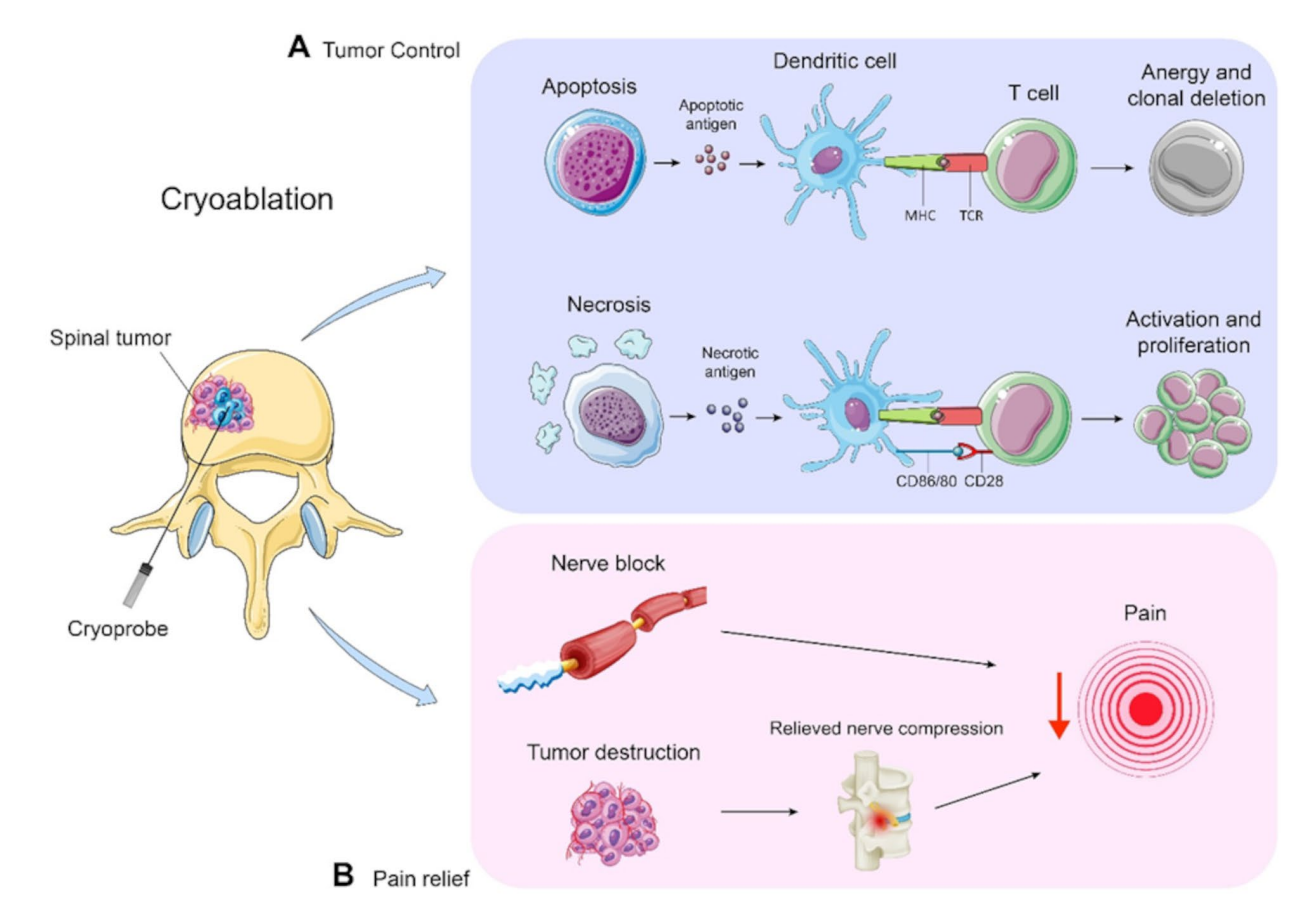
Schematic figure depicting cellular changes during the PCA procedure. This figure was generated from adopted figures provided by Smart Servier Medical Art (https://smart.servier.com ), which is licensed under a CC BY 4.0

Our meta-analysis demonstrated a local tumor control rate of 70% with PCA, comparable to the 60–100% range reported in a previous study [28]. In addition to direct tumor destruction, PCA may induce anti-tumor and pro-inflammatory effects enhancing immune responses by releasing tumor-specific antigens and damage-related molecular patterns (DAMPs). This process can activate dendritic cells, promoting CD4 + and CD8 + T lymphocyte responses and increasing production of pro-inflammatory cytokines like IL-1β, IL-6, IL-8, and IFN-γ (Fig. 2) [34].

The meta-regression analysis showed that tumors located in the vertebral body and older age were associated with lower local control rates. The reduced local tumor control in older patients may be due to more advanced stages of the disease, a diminished capacity to undergo extensive ablation, and a weakened anti-tumor response. The association between poorer outcomes and tumors located in the vertebral body is likely due to the increased risk of epidural involvement [35]. Tumors that invade the epidural space are typically more advanced and invasive [36]. Their proximity to the spinal cord and nerve roots often necessitates more conservative ablation margins, which can further reduce local tumor control rates [32].

This study found that PCA resulted in a 3.7-point reduction in pain on the first day after surgery and an additional 1.3-point reduction after one month. The short-term relief could be attributed to the analgesic effects of cryoablation as periosteal and bone innervation may be ablated during the procedure, whereas the mid‑term reduction is likely driven primarily by tumor destruction [37]. Because so few studies report quality of life both before and after PCA, we were unable to perform a meta‑analysis on this outcome. However, one previous investigation in patients with spinal metastases treated by RT found that pain response—defined as at least a 2‑point reduction on the NRS or a pain score of zero at the treated site—was significantly associated with improvements in the physical‑function domain of the EORTC QLQ‑C30 [38]. Accordingly, the 4.7‑point average reduction in pain score observed in our study likely translates into a meaningful enhancement of patients’ quality of life.

Previous studies found that tumor histology and size significantly influence the suitability and outcomes of PCA. For example, in primarily osteolytic lesions, the ice ball is easily visualized, enabling accurate assessment of ablation adequacy. However, in primarily osteoblastic lesions, the intraosseous ice ball is less distinct, which can increase the risk of injury to adjacent structures [13]. Furthermore, radioresistant spinal metastases, such as those arising from renal cell carcinoma, hepatocellular carcinoma, colorectal carcinoma, and non-small cell lung carcinoma, often respond poorly to RT but may benefit from PCA as an alternative treatment option. Smaller tumors (≤ 3–4 cm) that require fewer cryoprobes for ablation generally demonstrate better treatment responses and lower complication rates following PCA [39–41]. Additionally, studies have shown that patients younger than 70 years with favorable Eastern Cooperative Oncology Group (ECOG) performance scores (≤ 2) have a reduced risk of mortality and complications after PCA [41].

### Complications

PCA has a 2.0% major and 4.8% minor complication rate, mainly due to thermal injuries to nerves and the spinal cord. Nervous tissue is highly sensitive to temperature extremes, and the ice ball used in PCA can penetrate cortical bone, potentially damaging adjacent neural structures [42].

Protective measures help reduce these risks, with hydro-dissection or carbo-dissection combined with thermal monitoring being the most commonly used due to their ease of implementation through the same percutaneous access. More complex techniques like neural monitoring are reserved for high-risk tumors with large sizes or spinal canal invasion [18]. However, the current study was limited by the small number of articles reporting the use of protective measures, preventing direct comparisons of complication rates between those that employed these techniques and those that did not. Further research is needed to determine the most cost-effective protective measure for spinal PCA to minimize thermal injuries.

### Comparison

RT is essential in treating spinal tumors, using high-energy radiation to destroy or inhibit cancer cell growth [43]. A systematic review found that SBRT achieved a 36% complete pain response and a 94% 1-year local tumor control rate [44]. A study of 175 patients with painful bone metastases compared PCA, RT, and combined therapy. The combination resulted in significantly higher complete response rates than either alone, while PCA alone was more effective than RT (32% vs. 11.2%, *p* = 0.018) [45]. Regarding cost-effectiveness, PCA is considered a viable alternative to RT reirradiation for recurrent pain, though no strategy using initial PCA proved more cost-effective than RT [46]. RT is less effective against radioresistant tumors, such as those from the renal, thyroid, and gastrointestinal systems, as well as melanoma and sarcoma, which show higher local control failure rates compared to radiosensitive tumors like prostate cancer [47]. Conversely, PCA appears independent of tumor histology in achieving local tumor control, suggesting a broader potential application in pain management [48]. Furthermore, radiation-induced inflammation and fibrosis can obscure radiographic differentiation between residual tumor tissue and treatment-related remodeling in patients undergoing RT for spinal metastases [49]. In contrast, cryoablation produces well-demarcated ablation zones with minimal periprocedural edema [50]. Additionally, while RT is limited by cumulative dose thresholds that often preclude retreatment [51], cryoablation is a repeatable modality without concerns of dose accumulation. This is especially advantageous in cases of spinal metastases, where patients may require multiple interventions for multifocal or recurrent disease.

PCA has some advantages over RFA, including the ability to use multiple probes for overlapping ablation zones and effectiveness in treating osteoblastic metastases, where RFA may fail due to high impedance [52, 53]. Additionally, a previous systematic review study reported that the rate of complications was about 16% following RFA compared to 6.8% found for PCA in our study [54]. This may be because PCA’s freeze-induced injury is less harmful to neurovascular structures than heat-based methods [12]. RFA with cementoplasty offers the advantage of a short procedure time, with ablation lasting 1.5 to 9 min [55, 56] and an average total procedure duration of 40 to 60 min [57, 58]. Another advantage of RFA is the ability to inject cement immediately after ablation [59], whereas cryoablation’s iceball must thaw first—a process that is often lengthy and unpredictable.

MWA is a minimally invasive technique that delivers electromagnetic microwaves to tumorous tissue. A previous systematic review reported local tumor control rates exceeding 80% across multiple studies [60]. MWA offers several advantages, including reduced susceptibility to tissue impedance variations and perfusion-mediated cooling effects, as well as the potential to achieve higher intratumoral temperatures [60]. However, it also presents unique challenges in spinal applications. These include poorly defined ablation margins on imaging and a heightened risk of neural injury due to the rapid, high-power energy delivery near sensitive neural structures [61]. One study found that the complication rate for MWA was 27.4%, compared to 10.9% for patients undergoing RFA [61].

These findings suggest that PCA offers certain advantages over RFA and RT for the treatment of spinal metastases. However, further studies directly comparing these modalities are necessary to confirm and better understand their relative benefits.

### Limitations

This study has several limitations. This review used only PubMed and Embase, limiting broader coverage. Although sources like Cochrane Library and clinical trial registries were excluded, the number of potentially missed studies is likely minimal. The inclusion of articles addressing markedly different clinical indications, which may introduce substantial confounding bias. The retrospective design of the included studies and the resultant inability to account for key variables—such as prior treatments, comorbidities, and performance status—introduces potential unmeasured confounders that may have influenced the outcomes. The analysis was restricted by the small number of included studies, all of which were retrospective with small sample sizes, potentially introducing selection bias and reducing statistical power. Significant heterogeneity existed among studies regarding interventional approaches, patient selection criteria, protective measures, and imaging modalities affecting the generalizability of our findings. Some patients were included in multiple studies without specification (for example, two-thirds of the population in the study by Cazzato et al. [18] had previously been reported by Autrusseau et al. [20] and Tomasian et al. [13]), leading to potential data overlap and skewed results. Variations in follow-up durations may have influenced reported tumor control and pain outcomes. Differences in pain assessment methods, with some studies using the VAS and others the NRS, could introduce bias due to their subjective nature and cultural influences. Additionally, most patients had undergone prior treatments, such as chemotherapy or RT, which may have affected the efficacy and safety outcomes of PCA. These factors limit the generalizability of the findings and highlight the need for more standardized and prospective research.

## Conclusion

This systematic review suggests that PCA could be a viable treatment for spinal metastases in certain patients. Among 229 patients, PCA achieved a 70.5% local tumor control rate and significantly reduced pain scores from 6.98 pre-operatively to 2.62 at the last follow-up (average 12.5 months). PCA’s minimally invasive nature, coupled with a low complication rate of 2% for major events and 4.8% for minor ones, underscores its safety. PCA shows promise in managing spinal metastases, offering pain relief and local tumor control with minimal invasiveness. However, well‑designed randomized controlled trials are required to compare its efficacy, safety, and long‑term outcomes directly against current standard‑of‑care therapies.

## Electronic supplementary material

Below is the link to the electronic supplementary material.

Supplementary Material 1

## Data Availability

No datasets were generated or analysed during the current study.
