# Supplementary material for: Percutaneous cryoablation in the management of spinal metastases: a comprehensive systematic review and meta-analysis
Source: J Neurooncol. 2025 May 13;174(2):303–14. doi: 10.1007/s11060-025-05064-3 (PMC12208987; doi:10.1007/s11060-025-05064-3)
Supplement: Supplementary file 1 — Supplementary Material 1 [file 11060_2025_5064_MOESM1_ESM.docx]

| **Supplementary Table 1.** Search strategy and results. | | | |
| --- | --- | --- | --- |
| **PubMed** | | | |
| Date | Number of results | Search string | Row |
| July 13, 2024 | 122,723 | (((((((Cryoablation[Title/Abstract]) OR (Cryoablative[Title/Abstract])) OR (Cryo*[Title/Abstract])) OR (cryogenic[Title/Abstract])) OR (cryocoag*[Title/Abstract])) OR (Cryosurg*[Title/Abstract])) OR ("Cryosurgery"[MeSH])) | 1 |
| July 13, 2024 | 656,353 | (((Spine[Title/Abstract]) OR (Spinal[Title/Abstract])) OR (Vertebra*[Title/Abstract])) OR ("Spine"[Mesh]) | 2 |
| July 13, 2024 | 1,431 | #1 AND #2 | 3 |
| **Embase** | | | |
| July 13, 2024 | 170,306 | 'cryoablation'/exp OR cryoablative:ti,ab,kw OR cryo*:ti,ab,kw OR cryogenic:ti,ab,kw OR cryocoag*:ti,ab,kw OR cryosurg*:ti,ab,kw OR 'cryosurgery'/exp | 1 |
| July 13, 2024 | 780,593 | 'spine'/exp OR spinal:ti,ab,kw OR vertebra*:ti,ab,kw | 2 |
| July 13, 2024 | 2,245 | #1 AND #2 | 3 |

| **Supplementary Table 2.** Patient selection criteria of included studies. | |
| --- | --- |
| **Study** | **Patient selection criteria for PCA** |
| Cazzato et al., 2022 [1] | **Curative PCA** was considered for patients with oligometastatic disease (a small number of metastatic sites) or oligoprogressing disease (3–5 metastases that are not responding to systemic therapy).  **Palliative PCA** was considered for patients with painful spinal metastases that were refractory to or presented with recurrent pain following standard palliative therapy (e.g. analgesia and RT).  Patients with life expectancy < 1-month, irreversible coagulopathy, active sepsis, focal neurologic symptoms related to the target SM, mechanical instability assessed with the SINS score, and risks from anesthesia were not considered for PCA. |
| Gravel et al., 2022 [2] | NR |
| Autrusseau et al., 2021 [3] | **Indications:**  1.Patients seeking pain relief due to failure of or persistent pain despite standard treatments (analgesics, bisphosphonates, RT).  2. Patients seeking local tumor control due to an oligometastatic (less than five metastases) or oligoprogressing status (fewer than three metastases not responding to standard treatments).  **Contraindications:** short life expectancy (<1 month), irreversible coagulopathy, active sepsis, spinal cord compression, spinal instability assessed with the SINS score,18 or any other medical condition contraindicating conscious sedation and local anesthesia or general anesthesia.  PCA was preferentially used in case of large (>20 mm) or sclerotic tumors, or in case of tumors extending beyond the vertebral body. |
| Moses et al., 2020 [4] | **Inclusion**: Patients with a spinal tumor, including epidural tumor, and it was felt unsafe to proceed with stereotactic radiosurgery or if they had advanced metastatic disease and had previously undergone radiation and were deemed too unfit for traditional surgical intervention. Patients on active chemotherapy, where it was decided by the medical oncologist that treatment would need to stop for open surgery were considered for cryoablation. patients with lower KPS scores were favored for cryoablation, as it was often deemed unsafe for them to undergo open separation surgery.  **Exclusion:** Patients with acute neurological findings or an unstable spine. |
| Gravel et al., 2019 [5] | NR |
| Gallusser et al., 2019 [6] | NR |
| Motta et al., 2017 [7] | NR |
| McArthur et al., 2017 [8] | NR |
| Tomasian et al., 2016 [9] | Only patients with substantial pain as indicated by a score of at least 4 on a scale of 0 –10 were included. |
| Guenette et al., 2016 [10] | NR |
| Masala et al., 2013 [11] | **Inclusion:** Patients with a single vertebral level involvement, with lesion localized into the vertebral body without endospecal bone fragments and no epidural extension, refractory pain to pharmaceutical therapy from at least 3 months, and poor or absent response to RT or chemotherapy. |

| **Supplementary Table 3.** Additional details of included studies. | | | | | | |
| --- | --- | --- | --- | --- | --- | --- |
| **Study** | **Data collection years** | **Previous treatment** | **Protective measures** | **Anesthesia method** | **Adjuvant treatment** | **FU imaging modality** |
| Cazzato et al., 2022 [1] | 2008 - 2020 | NR | Hydrodissection (53 of 105 lesions), Theraml monitoring (48 lesions), Carbodissection (33 lesions), Neural monitoring (22 lesions) | GA: 51 of 79 procedures (64.6%) | RT (72 lesions) | Contrast-enhanced CT,  MR, and/or PET-CT |
| Gravel et al., 2022 [2] | 2011 - 2017 | NR | Neurological monitoring (3 procedures), Carbodissection (3 procedures) | All were under conscious sedation | NR | MRI (8–10 days after each procedure) |
| Autrusseau et al., 2021 [3] | 2008 - 2020 | NR | Hydrodissection (28/40 procedures), Carbodissection (12/40 procedures), Thermal monitoring (36/55 procedures), SEP (1/55 procedures) | GA: 42/50 procedures (84%); conscious sedation or LA: 8/50 procedures (16%) | Vertebral augmentation (20/55 procedures) | Contrast-enhanced MRI (1-month and thereafter at the discretion of the referring physician) |
| Moses et al., 2020 [4] | 2015 - 2018 | Chemotherapy and RT (9 pts), Chemotherapy (4 pts), Surgery and RT (1 pt) | NR | GA: 14 of 14 pts (100%) | SRS (5 pts), Surgery (2 pts) | NR |
| Gravel et al., 2019 [5] | 2011 - 2017 | Systemic treatment (32 pts), RT (1 pt) | Carbodissection (35 procedures) | GA: 10 of 41 procedures (24.4%), conscious sedation: 31 of 41 procedures (75.6%) | Percutaneous vertebroplasty (25 pts) | CT and/or MRI, and/or abnormal  18F-FDG PET/CT uptake (at 1-year FU) |
| Gallusser et al., 2019 [6] | 2011 - 2016 | Surgery, Chemotherapy, and RT (3 pts), Surgery, Chemotherapy, and Hormonotherapy (1 pt) | NR | NR | Kyphoplasty (1 pt) | MRI and/or PET/CT |
| Motta et al., 2017 [7] | 2013 - 2016 | Chemotherapy and RT (9 pts), SRT (1 pt) | NR | LA: 11 of 11 pts (100%) | NR | NR |
| McArthur et al., 2017 [8] | 2005 - 2011 | Chemotherapy (3 pts) | NR | GA: 3 of 3 pts (100%) | NR | CT (4-6 weeks after the procedure) |
| Tomasian et al., 2016 [9] | NR | RT (8 of 31 lesions) | Neuroforaminal thermal monitoring (14) | GA: 3 of 31 lesions (9.7%); Conscious sedation: 28 of 31 procedures (90.3%) | Vertebroplasty (1 pt) | MRI and  PET/CT (1–24 months after the procedure) |
| Guenette et al., 2016 [10] | 2014 - 2015 | NR | NR | GA: 3 of 3 pts (100%) | NR | CT and/or MRI (1-22 months after the procedure) |
| Masala et al., 2013 [11] | 2010 - 2011 | RT with or without Chemotherapy (23 pts) | NR | LA: 20 of 23 pts (87%); Conscious sedation: 3 of 23 pts (13%) | Vertebroplasty (23 pts) | NR |
| Abbreviations: NR: Not reported; RT: Radiation therapy; GA: General anesthesia; LA: Local anesthesia; SRS: Stereotactic radiosurgery | | | | | | |

| **Supplementary Table 4**. Risk of bias assessment using JBI Critical Appraisal Checklist for Case Series. | | | | | | | | | | | |
| --- | --- | --- | --- | --- | --- | --- | --- | --- | --- | --- | --- |
| **Author, year** | **Q1** | **Q2** | **Q3** | **Q4** | **Q5** | **Q6** | **Q7** | **Q8** | **Q9** | **Q10** | **Overall** |
| Cazzato, 2022 [1] | Y | Y | Y | Y | Y | Y | Y | Y | Y | Y | Low |
| Gravel, 2022 [2] | N | Y | Y | N | N | N | N | Y | N | N | High |
| Autrusseau, 2021 [3] | Y | Y | Y | Y | Y | Y | Y | Y | Y | Y | Low |
| Moses, 2020 [4] | Y | Y | Y | Y | Y | Y | Y | Y | Y | Y | Low |
| Gravel, 2019 [5] | Y | Y | Y | Y | Y | Y | Y | Y | Y | Y | Low |
| Gallusser, 2019 [6] | Y | Y | Y | Y | Y | Y | Y | Y | Y | Y | Low |
| Motta, 2017 [7] | Y | Y | Y | Y | Y | Y | Y | Y | Y | Y | Low |
| McArthur, 2017 [8] | Y | Y | Y | Y | Y | Y | Y | Y | Y | Y | Low |
| Tomasian, 2016 [9] | Y | Y | Y | Y | Y | Y | Y | Y | Y | Y | Low |
| Guenette, 2016 [10] | Y | Y | Y | Y | Y | Y | Y | N | Y | Y | High |
| Masala, 2013 [11] | Y | Y | Y | Y | Y | Y | Y | Y | Y | Y | Low |
| Abbreviations: Y: Yes; N: No; U: Unclear.  Questions: Q1: Were there clear criteria for inclusion in the case series? Q2: Was the condition measured in a standard, reliable way for all participants included in the case series?  Q3: Were valid methods used for identification of the condition for all participants included in the case series?  Q4: Did the case series have consecutive inclusion of participants?  Q5: Did the case series have complete inclusion of participants?  Q6: Was there clear reporting of the demographics of the participants in the study?  Q7: Was there clear reporting of clinical information of the participants?  Q8: Were the outcomes or follow up results of cases clearly reported?  Q9: Was there clear reporting of the presenting site(s)/clinic(s) demographic information?  Q10: Was statistical analysis appropriate? | | | | | | | | | | | |


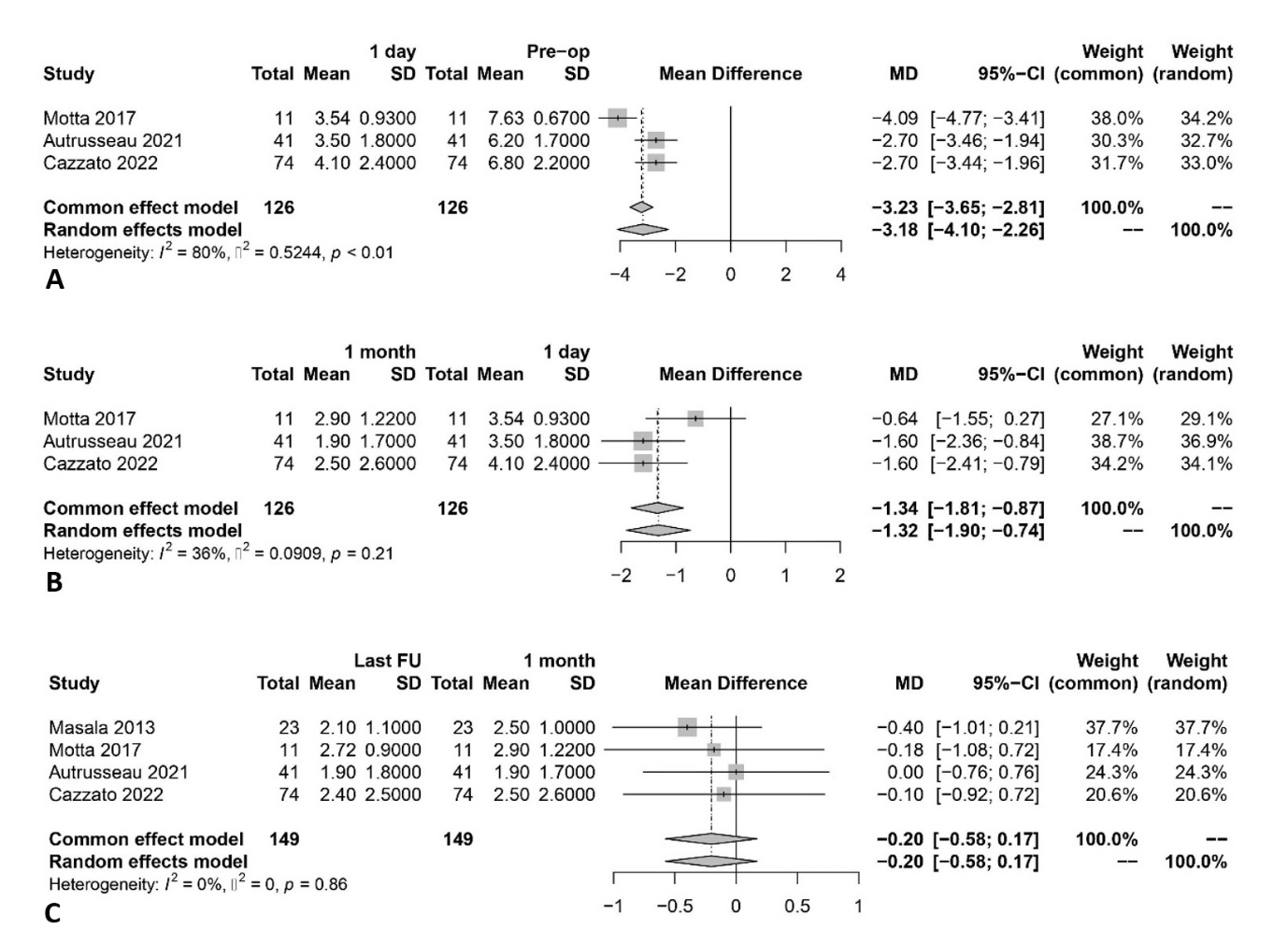


**Supplementary Figure 1.** Forest plot comparing the pain severity scores in the pre-operative. One day (A). One month (B). and last follow-up (C) after the procedure.


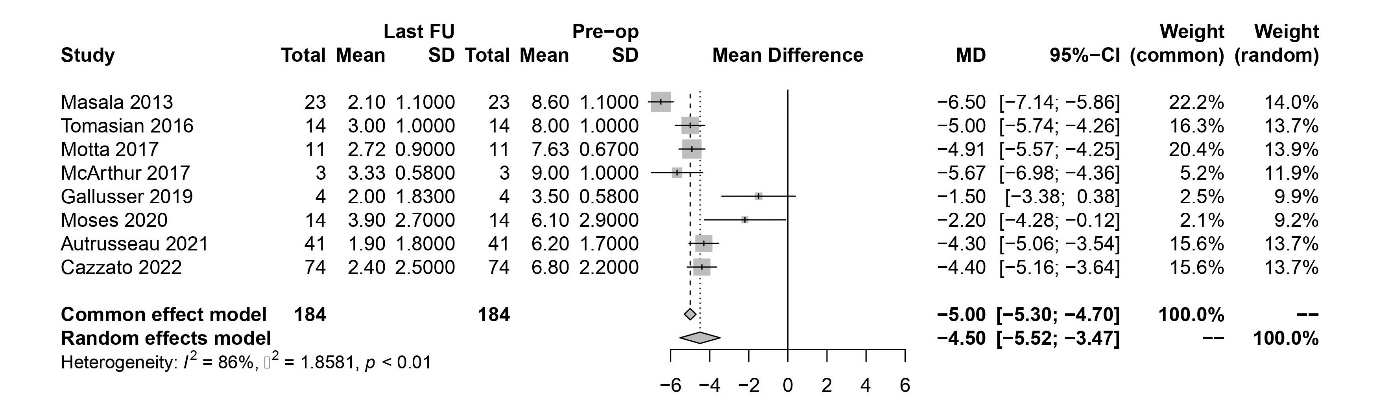


**Supplementary Figure 2.** Forest plot revealing the mean difference in pain scale from preoperative to last follow-up.

**References**

1. Cazzato RL, Jennings JW, Autrusseau PA, De Marini P, Auloge P, Tomasian A, Garnon J, Gangi A (2022) Percutaneous image-guided cryoablation of spinal metastases: over 10-year experience in two academic centers. Eur Radiol 32: 4137-4146 doi:10.1007/s00330-021-08477-6

2. Gravel G, Roussel A, Mellot F (2022) Anterior epidural carbon dioxide dissection during spine cryoablation. Jpn J Radiol 40: 103-105 doi:10.1007/s11604-021-01171-z

3. Autrusseau PA, Cazzato RL, De Marini P, Auloge P, Koch G, Dalili D, Weiss J, Mayer T, Garnon J, Gangi A (2021) Pain relief and local tumour control following percutaneous image-guided cryoablation for spine metastasis: a 12-year single-centre experience. Clin Radiol 76: 674-680 doi:10.1016/j.crad.2021.05.013

4. Moses ZB, Lee TC, Huang KT, Guenette JP, Chi JH (2020) MRI-guided cryoablation for metastatic spine disease: intermediate-term clinical outcomes in 14 consecutive patients. J Neurosurg Spine: 1-6 doi:10.3171/2019.11.Spine19808

5. Gravel G, Tselikas L, Moulin B, Yevich S, Baudin E, Hakime A, Moalla S, Mihoubi F, Balleyguier C, de Baere T, Deschamps F (2019) Early detection with MRI of incomplete treatment of spine metastases after percutaneous cryoablation. Eur Radiol 29: 5655-5663 doi:10.1007/s00330-019-06040-y

6. Gallusser N, Goetti P, Becce F, Vauclair F, Rüdiger HA, Bize PE, Cherix S (2019) Percutaneous image-guided cryoablation of painful bone metastases: A single institution experience. Orthop Traumatol Surg Res 105: 369-374 doi:10.1016/j.otsr.2019.01.001

7. Motta A, Caltabiano G, Palmucci S, Failla G, Basile A (2017) Feasibility of percutaneous cryoablation of vertebral metastases under local anaesthesia in ASAIII patients. Eur J Radiol 95: 13-17 doi:10.1016/j.ejrad.2017.07.011

8. McArthur TA, Narducci CA, Lander PH, Lopez-Ben R (2017) Percutane Image-Guided Cryoablation of Painful Osseous Metastases: A Retrospective Single-Center Review. Curr Probl Diagn Radiol 46: 282-287 doi:10.1067/j.cpradiol.2016.11.007

9. Tomasian A, Wallace A, Northrup B, Hillen TJ, Jennings JW (2016) Spine Cryoablation: Pain Palliation and Local Tumor Control for Vertebral Metastases. AJNR Am J Neuroradiol 37: 189-195 doi:10.3174/ajnr.A4521

10. Guenette JP, Tuncali K, Himes N, Tatli S, Lee TC (2016) Spine Cryoablation: A Multimodality Image-Guided Approach for Tumors Adjacent to Major Neural Elements. AJNR Am J Neuroradiol 37: 2396-2399 doi:10.3174/ajnr.A4923

11. Masala S, Chiocchi M, Taglieri A, Bindi A, Nezzo M, De Vivo D, Simonetti G (2013) Combined use of percutaneous cryoablation and vertebroplasty with 3D rotational angiograph in treatment of single vertebral metastasis: comparison with vertebroplasty. Neuroradiology 55: 193-200 doi:10.1007/s00234-012-1096-7
